# TOBACCO IN HOTELS: A STUDY OF NICOTINE EXPOSURE AND RESIDENT RISK

**DOI:** 10.1093/geroni/igad104.1911

**Published:** 2023-12-21

**Authors:** Terri Lewinson, Abhirupta Dasgupta, James Murphey

**Affiliations:** Geisel School of Medicine/ Dartmouth College, Lebanon, New Hampshire, United States; Geisel School of Medicine, Dartmouth College, Hanover, New Hampshire, United States; Geisel School of Medicine, Dartmouth College, Hanover, New Hampshire, United States

## Abstract

Secondhand smoke (SHS) exposure exacts considerable health and financial impact on thousands of Americans yearly. One in four nonsmokers (about 58 million people) is routinely exposed to SHS, primarily at home. There is wide variability in smoke-free home policy adoption, leading to sociodemographic disparities in tobacco exposure and adverse health effects among low-income renters. Although there is no safe level of tobacco smoke exposure, varying perceptions about exposure effects and risks contribute to inconsistent use of harm-reduction strategies such as smoke-free home policies in private dwellings. Therefore, we surveyed and interviewed low-income extended-stay hotel renters (n=77) to learn about their perceptions of tobacco exposure. We also measured participants’ willingness to self-monitor these risks and adopt at-home smoke-free interventions. Most survey participants identified as nonsmokers (56.8%) exposed to tobacco smoke regularly. However, both nonsmokers and smokers preferred smoke-free rooms and indicated they would likely participate in personal health research by sharing air and biosamples to measure their exposure. We will present survey results of tobacco exposure knowledge and attitudes among long-term renters in Metropolitan Atlanta extended-stay hotels, perceptions of exposure risk, and self-efficacy beliefs. From our interview findings, we share renter and hotel manager readiness perceptions for implementing at-home smoke-free interventions. Lastly, we will share the results of integrating community-engaged partnerships with local organizations and respondent-driven sampling methods to build trust and aid renter recruitment.

